# Multi-district molecular surveillance of dengue virus in Pakistan reveals serotype cycling and recurrent DENV-2 dominance, 2021–2025

**DOI:** 10.1371/journal.pone.0353487

**Published:** 2026-07-23

**Authors:** Massab Umair, Rabia Hakim, Zunera Jamal, Qasim Ali, Muhammad Salman

**Affiliations:** Department of Virology, National Institute of Health, Islamabad, Pakistan; Defense Threat Reduction Agency, UNITED STATES OF AMERICA

## Abstract

Dengue virus (DENV) remains a major public health concern in Pakistan, with recurrent seasonal outbreaks and shifting serotype patterns. Understanding temporal and geographic trends in circulating serotypes is essential for improving surveillance and outbreak preparedness. This study aimed to characterize the spatio-temporal distribution and serotype dynamics of DENV detected through molecular surveillance in Pakistan between 2021 and 2025. NS1-positive dengue cases were tested at the National Institute of Health (NIH), Islamabad, using real-time reverse transcription polymerase chain reaction (qRT-PCR) for serotyping. Demographic, temporal, and geographic data were analyzed to assess the distribution of DENV infections and circulating serotypes over the study period. Among 1,644 NS1-positive dengue cases tested, 1,162 were confirmed by qRT-PCR. DENV-2 was the predominant serotype overall, accounting for 78.4% of confirmed infections, followed by DENV-1 (21.4%) and DENV-3 (0.09%). Temporal analysis showed that DENV-2 predominated during 2021 and 2022, was temporarily replaced by DENV-1 in 2023, and re-emerged as the dominant serotype in 2024 and 2025. Dengue transmission demonstrated strong seasonal patterns, with most cases occurring during the post-monsoon months of September and October. Gender-Age distribution showed majority of cases in males (66%), particularly those aged 21–40 years. Geographically, most confirmed cases were reported from Rawalpindi and Islamabad, reflecting the primary referral catchment of the surveillance system, while additional cases were detected from several other districts, including Peshawar, Nowshera, Mansehra, and Karachi. These findings highlight dynamic shifts in DENV serotype circulation in Pakistan and emphasize the need for sustained molecular surveillance to guide public health responses.

## Introduction

Dengue is a rapidly growing and re-emerging viral epidemic across many tropical and subtropical nations worldwide [1]. Dengue virus (DENV), which belongs to family *Flaviviridae* within the genus *Flavivirus*, is a single stranded positive-sense RNA virus that spreads through the bite of an infected female mosquito from the family *Aedes Aegypti* and *Aedes albopictus* [2]. DENV exists as four serotypes (DENV-1 to DENV-4) that share approximately 65–70% nucleotide identity but are antigenically and phylogenetically distinct, with DENV-1 and DENV-3 being more closely related while DENV-4 is the most genetically divergent. [3]. Each serotype is classified into genotypes based on variations in the envelope (E) protein, as follows: DENV-1 (I–VI), DENV-2 (Asian I/II, Asian/American, American, cosmopolitan, sylvatic, or 1-VI), DENV-3 (I–V), and DENV-4 (Asian I/II, Asian/American, American, cosmopolitan, sylvatic, or I-IV) [4]. Numerous studies have shown that the Southeast Asian genotype of DENV-2 can infect humans more severely than the indigenous American genotype due to higher ability to infect new mosquitoes and proliferate in human cells [2]. Clinically, dengue infection can range from mild to severe dengue fever with hemorrhagic manifestations. Multiple factors can contribute to dengue hemorrhagic fever (DHF) such as co-infections, age, viral load, immune enhancement and infecting serotype or genotype [5].

According to the World Health Organization (WHO), half of the world population is now at risk because of the dramatic growth of DENV infection incidence in recent decades. DENV infects approximately 100–400 million individuals each year throughout the world [6]. Serological evidence of DENV infection in Pakistan dates back to 1968, while the first DHF outbreak was reported in Karachi in 1994 [7]. A major outbreak involving DENV-1, DENV-2, and DENV-3 occurred in Hub, Balochistan, in 1995, followed by a DENV-2 outbreak in 2003 causing 3500 cases and 18 deaths in Haripur, Nowshera, and Khushab. Dengue re-emerged in 2005 with 500 cases and 13 deaths in Islamabad and Karachi [8]. Since then, recurrent outbreaks have affected Khyber Pakhtunkhwa, Punjab, and Sindh, with the largest burden recorded in Punjab during 2011 (286,857 suspected cases, 25,932 confirmed, and 62 deaths) [9]. A major epidemic occurred in Swat and Mansehra during 2013–2014, including 8546 cases and 33 deaths in Swat in 2013 (estimated ~6000 cases), with DENV-1, DENV-2, and DENV-3 circulating [9]. In 2015, DENV-3 caused an outbreak in Malakand and 9899 cases were reported nationwide, while Sindh recorded the highest cases in 2016. Reported cases fluctuated between 2017 and 2021 (22,938 in 2017; 3204 in 2018; 24,547 in 2019; 3442 in 2020), followed by a major surge in 2021 with 48,906 confirmed cases in Lahore and the twin cities (Rawalpindi and Islamabad) [10]. In 2022, severe flooding and heavy rainfall intensified transmission, leading to 21,685 cases and 350 deaths, with 83% of cases distributed across Sindh (32%), Punjab including ICT (29%), and Balochistan (14%) [11]. Peshawar alone reported 9,490 laboratory-confirmed cases between June and December [12]. In 2023, Pakistan again recorded a high burden with 20,072 dengue cases [13]. As of December 2024, Pakistan reported approximately 20,000 dengue cases, including a sharp weekly increase of nearly 3000 cases in June. Balochistan recorded the highest number of cases (6958), followed by Punjab (5405), Khyber Pakhtunkhwa (3649), and Sindh (1167). Among urban centers, Islamabad reported 3,754 cases, while Rawalpindi recorded 2,143 cases [14].

Despite recurrent dengue outbreaks in Pakistan, comprehensive data on the temporal dynamics of circulating DENV serotypes remain limited. Most previous reports have been restricted to outbreak investigations or localized studies, providing only fragmented insights into serotype distribution across time and regions. While DENV-1, DENV-2, and DENV-3 have been reported in different parts of the country, systematic molecular surveillance describing recent serotype trends is scarce. Understanding the changing patterns of circulating serotypes is important because shifts in serotype dominance can influence transmission dynamics, outbreak magnitude, and disease severity in dengue-endemic settings. Therefore, this study aimed to characterize the temporal trends and geographic distribution of DENV serotypes detected through molecular surveillance between 2021 and 2025, providing updated insights into dengue epidemiology in Pakistan.

## Materials and methods

### Study design and sample collection

This study analyzed serum samples received at the Virology Department of the National Institute of Health Pakistan, Islamabad, between January 2021 and October 2025. The laboratory functions as a national reference center for arboviral diagnostics and receives clinical samples from hospitals across Pakistan for confirmatory testing and serotype identification. Suspected dengue cases were initially screened using dengue NS1 antigen assays at referring hospitals. NS1-positive samples were subsequently referred to NIH Pakistan, for serotype determination, as most referring hospitals lacked in-house molecular diagnostic capacity. The majority of samples originated from public sector tertiary care hospitals. The study represents a retrospective analysis of DENV samples submitted through passive laboratory-based surveillance. The geographic distribution of samples reflects referral patterns and may not represent the true distribution of dengue infections across Pakistan.

During the study period, a total of 1,644 serum samples from NS1-positive dengue patients were received for serotyping. Samples were submitted from multiple districts, including Rawalpindi (n = 916), Islamabad (n = 273), Peshawar (n = 128), Mansehra (n = 96), Nowshera (n = 81), Mardan (n = 22), Karachi (n = 20), Kech (n = 20), Attock (n = 16), Swat (n = 16), Muzaffarabad (n = 14), Chillas (n = 13), Khyber Agency (n = 11), Abbottabad (n = 7), Jhelum (n = 6), and Quetta (n = 5). Demographic, clinical, and hematological information were extracted from standardized case report forms accompanying each sample. Samples received under inappropriate conditions (e.g., leakage, improper storage, or inadequate packaging) (n = 53) were excluded from the analysis. Also, Clinical information was available for 604 patients, whereas hematological data was available for 598 cases. All qRT-PCR-confirmed cases were included in the serotype distribution analysis.

### Ethical approval

This study was approved by the Institutional Review Board (IRB) of the National Institute of Health Pakistan, Islamabad, Pakistan (F.1–5/RAPiD/2025–26/IRB-03). The study involved retrospective analysis of de-identified laboratory samples collected as part of routine dengue surveillance, and individual informed consent was waived by the IRB.

### Viral RNA extraction & serotype specific PCR amplification

Viral RNA was extracted from serum samples using the QIAamp Viral RNA Mini Kit (Qiagen, Hilden, Germany) according to the manufacturer’s protocol. Extracts were eluted in 60 μL of AVE buffer and stored at −80°C until use. Serotyping was performed using a modified four-plex real-time TaqMan RT-PCR assay based on the Centers for Disease Control and Prevention (CDC) protocol [15] were utilized. Each multiplex reaction, with a total volume of 25 µL, was carried out for all four DENV serotypes (DENV-1 to DENV-4) using an Invitrogen SuperScript™ III Platinum™ One-Step qRT-PCR Kit (Carlsbad, CA, USA) on Applied Biosystems QuantStudio 5 Real-Time PCR System. The cycling conditions were as follows: RT step 50°C for 10 minutes, initial denaturation at 95°C for 5 minutes and 45 cycles at 95°C for 15 seconds and at 60°C for 60 seconds.

### Statistical analysis

Descriptive statistics were used to assess the prevalence of clinical symptoms and hematological markers among confirmed dengue cases. Associations between DENV serotypes (DENV-1 and DENV-2) and clinical or hematological parameters were examined using Chi-square (χ²) test. DENV-3 (n = 1) was excluded from comparative analysis, and statistical analyses were performed using SPSS version 29 (IBM Corp., USA), with p < 0.05 considered significant.

## Results

### Sample characteristics and geographic distribution

**Fig 1:**
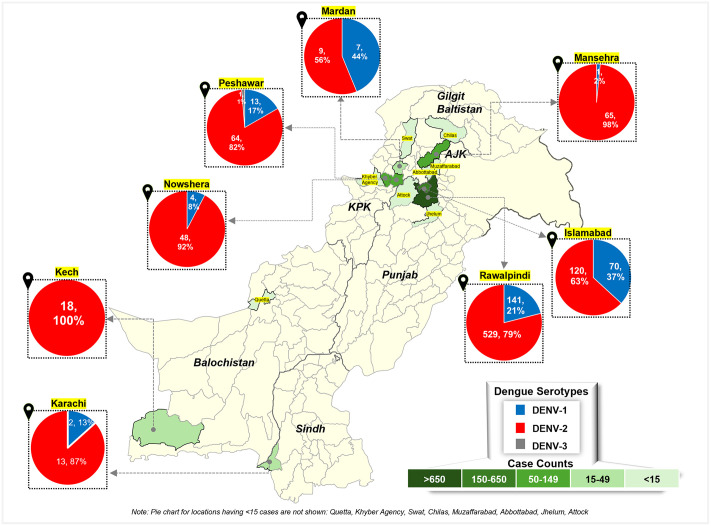
Geographical distribution of DENV-1, DENV-2 and DENV-3 positive samples in Pakistan during 2021–2025. Pie charts show the absolute number (n) and percentage distribution of DENV serotypes within each district, while the green color gradient indicates the total number of confirmed cases. The confirmed cases were highest in Rawalpindi (n = 670) and Islamabad (n = 190), followed by Peshawar (n = 78) and Mansehra (n = 66), with remaining districts showing lower case numbers. Samples were collected over different years within the study period; therefore, the figure reflects data from multiple years (see S1 Table). Map source: Wikimedia Commons (Pakistan districts SVG), released into the public domain by the copyright holder, Available at: https://commons.wikimedia.org/wiki/File:Map_of_Pakistan_%282018%29.svg.

During the study period (January 2021–October 2025), a total of 1,644 serum samples from NS1-positive dengue patients were received for serotyping, of which 1,162 (70.7%) were confirmed positive by qRT-PCR. The confirmed cases were distributed across multiple districts, with the highest numbers from Rawalpindi (n = 670; 57.6% of total positives) and Islamabad (n = 190; 16.3%), followed by Peshawar (n = 78; 6.6%), Mansehra (n = 66; 5.6%), Nowshera (n = 52; 4.4%), and Kech (n = 18; 1.6%), with fewer cases reported from Mardan (n = 16), Karachi (n = 15), Muzaffarabad (n = 10), Attock (n = 13), Swat (n = 8), Chillas (n = 8), Khyber Agency (n = 7), Jhelum (n = 5), Abbottabad (n = 3), and Quetta (n = 3) (Fig 1). The geographic distribution of confirmed cases reflects referral patterns from participating hospitals and may not represent the true population-level distribution of dengue infections. District-wise and year-wise sampling details are provided in the S1 Table.

### Temporal distribution of dengue cases

**Fig 2:**
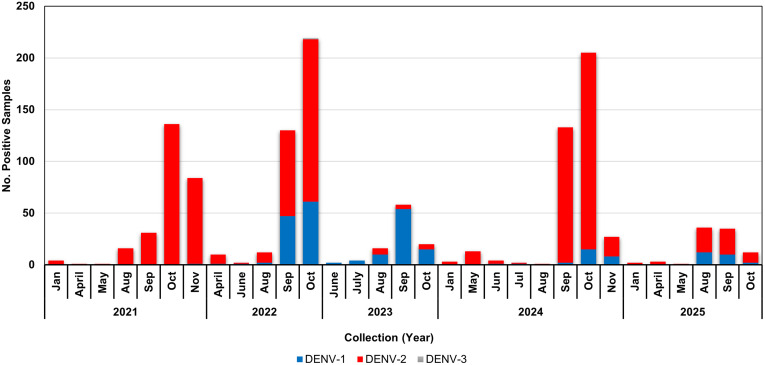
Monthly distribution of DENV-1, DENV-2 and DENV-3 positive samples during 2021-2025 in Pakistan.

Monthly analysis of confirmed dengue cases demonstrated a clear seasonal pattern, with most infections predominantly occurring during the post-monsoon period from August to October, peaking in October and September. Early-year cases from January to July accounted for less than 10% of the total. Cases typically roses in July, and declined toward the end of the year. Overall, more than 85% of cases were reported during the late monsoon and post-monsoon months, highlighting a brief but intense dengue transmission season (Fig 2).

### Dengue virus serotype distribution

The serotype distribution among 1,162 qRT-PCR confirmed dengue-positive samples demonstrated dominance of DENV-2 (78.5%; n = 912), followed by DENV-1 (21.4%; n = 249), and DENV-3 (0.09%; n = 1), while DENV-4 was not detected. DENV-2 predominated overall, accounting for 99.63% (n = 272) in 2021, 63.78% (n = 199) in 2022, 93.04% (n = 361) in 2024, and 73.03% (n = 65) in 2025. In 2023, DENV-1 gradually became dominant (85%; n = 85), while DENV-2 declined to 15.0%. DENV-3 remained sporadic, representing only single case in 2022 and was absent in other years. These results demonstrate dynamic temporal shifts in serotype circulation over time (Fig 2), while the detailed year-wise breakdown of cases is provided in Table 1.

**Table 1:** Year-wise distribution of dengue virus serotypes among qRT-PCR-confirmed cases (2021–2025) in Pakistan.

Year	DENV-1 n (%)	DENV-2 n (%)	DENV-3 n (%)	Total
2021	1 (0.37)	272 (99.63)	0 (0.00)	273
2022	112 (35.90)	199 (63.78)	1 (0.32)	312
2023	85 (85.00)	15 (15.00)	0 (0.00)	100
2024	27 (6.96)	361 (93.04)	0 (0.00)	388
2025	24 (26.97)	65 (73.03)	0 (0.00)	89
**Total**	**249 (21.43)**	**912 (78.49)**	**1 (0.09)**	**1,162**

### Age and gender distribution

**Fig 3:**
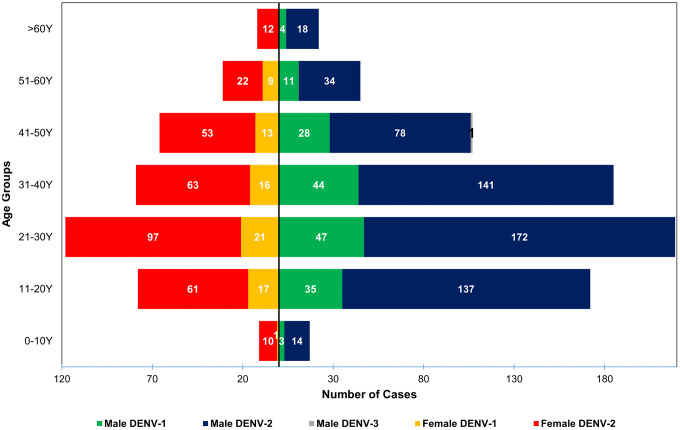
Age and gender distribution of DENV-1, DENV-2, and DENV-3 positive cases during 2021-2025 in Pakistan.

The distribution of confirmed dengue cases by age group, gender, and infecting serotype is presented in Fig 3. Overall, dengue infections were more frequently observed in males (66%) than females (34%). The highest number of cases occurred among young adults (21–30 years), representing 29% of total cases, followed by those aged 31–40 years (22.7%) and 41–50 years (14.8%). Lower numbers of cases were observed among children and older adults.

DENV-2 was the most frequently detected serotype across most age groups, particularly among adults aged 21–40 years. DENV-1 contributed a smaller proportion of infections across age groups, while DENV-3 infections were rare.

### Clinical and hematological characteristics

Clinical and hematological information was available for a subset of patients (clinical data: n = 604; hematological data: n = 598) (Table 2). Fever was the most frequently reported symptom (99.6%), followed by myalgia (98.0%) and backache (60.8%).

**Table 2:** Clinical and Hematological characteristics of Dengue patients.

Category	Parameter	Count (Percentage)	95% CI (%)	p-value (vs null)
**Clinical Symptoms**				
(Total n = 604)	Fever	602 (99.6%)	98.80–99.91	< 0.001
	Myalgia	591 (98.0%)	96.35–98.74	< 0.001
	Backache	367 (60.8%)	56.81–64.58	< 0.001
**Hematological Markers**				
(Total n = 598)	Platelet count ≤150,000	569 (95.2%)	93.12–96.60	< 0.001
	Platelet count >150,000	29 (4.8%)		(Not tested; low prevalence)
	Hematocrit Level <40	287 (48.0%)	44.01–52.00	0.32
	Hematocrit Level 41–45	146 (24.4%)	21.14–28.01	< 0.001
	Hematocrit Level >45	155 (25.9%)	22.57–29.58	< 0.001
	WBC count <4000	472 (78.9%)	75.48–82.01	< 0.001
	WBC count ≥4000	126 (21.1%)		(Not tested; low prevalence)

Hematological abnormalities were common among confirmed dengue cases. Thrombocytopenia (platelet count ≤150,000/µL) was observed in 95.2% of patients, while leukopenia (white blood cell count <4000/µL) was detected in 78.9%. Hematocrit values were <40% in 48.0% of patients, 41–45% in 24.4%, and >45% in 25.9%.

### Chi-square association between serotype and clinical features

As shown in Table 3, Chi-Square association analyses of qRT-PCR-confirmed DENV-1 (n = 129) and DENV-2 (n = 256) cases with available clinical or hematological information showed that fever, myalgia, and backache were not significantly associated with serotypes (p > 0.05). Among patients with hematological data (DENV-1: n = 105; DENV-2: n = 325), thrombocytopenia and leukopenia were common in both serotypes but were not significantly (p > 0.05) associated. Therefore, the analysis does not support a statistically significant association between serotype and the assessed clinical or hematological features.

**Table 3:** Association between Dengue Virus Serotypes and Clinical/Hematological Characteristics.

Variable	DENV-1 n (%)	DENV-2 n (%)	χ²	p-value
**Clinical symptoms**	**Total n = 129**	**Total n = 256)**		
Fever	129 (100.0%)	254 (99.2%)	0.07	0.798
Myalgia	126 (97.7%)	246 (96.1%)	0.26	0.609
Backache	74 (57.4%)	161 (62.9%)	0.88	0.348
**Hematological markers**	**Total n = 105**	**Total n = 325**		
Platelet count ≤150,000/µL	98 (93.3%)	316 (97.2%)	2.36	0.124
WBC count <4,000/µL	65 (61.9%)	181 (55.7%)	1.01	0.315

## Discussion

DENV remains a major public health challenge in Pakistan, with recurrent outbreaks since 1994. This multi-district molecular surveillance study (2021–2025) demonstrates sustained DENV-2 dominance in 2021–2022 and 2024–2025, with a transient shift to DENV-1 in 2023. Such serotype dynamics may influence disease severity, as DENV-2 has been associated with more severe outcomes, particularly in secondary infections [16]. Consistently, DENV-2–dominant years in Pakistan showed higher disease burden, including 48,906 cases with 183 deaths in 2021 [17], and 75,450 cases with 136 deaths in 2022 [18], whereas the DENV-1–dominant year 2023 reported comparatively lower cases (~20,072) [4]. DENV-2 re-emerged in 2024 with ~28,427 cases, with Balochistan (6,958 cases) most affected followed by Punjab (5405), Khyber Pakhtunkhwa (3649), and Sindh (1167) [14,19]. The absence of DENV-4 and mixed serotype infections in our dataset may indicate limited circulation of these variants; however, it may also reflect constraints of passive, referral-based surveillance. Although limited mortality data, particularly for 2025, restrict definitive conclusions, our findings suggest an ecological association between serotype replacement and outbreak severity rather than direct causation, while emphasizing the importance of continued molecular and genomic surveillance to detect emerging serotypes and strengthen outbreak preparedness.

A difference was observed between NS1 antigen positivity (n = 1,644) and qRT-PCR confirmation (n = 1,162; 70.7%), reflecting expected variability between antigen-based and molecular assays. NS1 detection is more sensitive in early infection, whereas qRT-PCR depends on viral RNA levels that may decline with disease progression. This discrepancy may also reflect the widespread use of NS1 rapid diagnostic tests in Pakistan, which are commonly used as initial screening tools but may have lower specificity compared to real-time PCR, potentially contributing to false-positive results [20]. Importantly, serotype distribution analysis was restricted to qRT-PCR–confirmed cases, thereby ensuring that only molecularly validated infections were included.

The geographic distribution of cases in our surveillance dataset shows that a substantial proportion of dengue infections originated from the twin cities of Rawalpindi and Islamabad during 2021–2025. Previous studies have similarly identified these urban centers as major dengue transmission hotspots in Pakistan [21]. Urban environmental factors such as high population density, favorable temperature and rainfall patterns, and rapid urban expansion contribute significantly to dengue transmission in major Pakistani cities including Islamabad, Rawalpindi, Lahore, and Karachi [10]. The Islamabad Rawalpindi metropolitan region hosts a population exceeding 5.7 million, creating densely populated environments that facilitate interactions between human hosts and *Aedes* mosquito vectors [22]. These demographic and ecological conditions support sustained dengue transmission where mosquito breeding sites and human–vector contact are common. Another factor contributing to the increasing dengue cases in the Rawalpindi and Islamabad is the intense daily movement of people between Rawalpindi and Islamabad and surrounding districts [23]. Human mobility is a key driver of dengue transmission, as infected individuals can introduce the virus into new areas where local *Aedes* mosquito populations sustain transmission. Studies using mobility data in Pakistan have shown that travel patterns strongly influence the spatial spread and timing of dengue outbreaks. Frequent commuting between the interconnected twin cities (Rawalpindi and Islamabad), along with connectivity to nearby districts such as Attock, Nowshera, Mansehra, and Peshawar, likely facilitates continuous viral circulation and regional spread. The continued detection of DENV-2 in the Rawalpindi and Islamabad across multiple years in our surveillance therefore suggests the possibility of localized endemic circulation rather than repeated independent introductions. In contrast, the temporary replacement by DENV-1 observed in 2023 may represent a typical serotype replacement event driven by population immunity against the previously dominant serotype. Such cyclical shifts between DENV-1 and DENV-2 have been documented in several dengue-endemic countries and are considered a hallmark of hyper endemic dengue transmission where multiple serotypes co-circulate over time [24]. Together, these findings highlight the importance of sustained molecular surveillance in major urban transmission hubs such as Rawalpindi and Islamabad to monitor serotype transitions and anticipate potential changes in outbreak dynamics.

The temporal shifts in dominant serotypes observed in this study are consistent with patterns reported across several dengue-endemic regions, including our previous genomic study covering 2024 outbreak in Pakistan [24,25]. Cyclical replacement between DENV-1 and DENV-2 has been frequently documented in South and Southeast Asia, including neighboring countries such as India, Bangladesh, and Sri Lanka [26–28]. In these settings, alternating serotype predominance has been associated with changing population immunity, viral fitness, and ecological conditions that influence vector transmission [29]. Similar dynamics have also been reported in parts of Southeast Asia and Latin America, where dengue epidemiology is characterized by periodic serotype replacement and the co-circulation of multiple serotypes [30]. The patterns observed in Pakistan therefore appear to align with broader regional dengue transmission dynamics.

Although DENV-2 has been associated with increased disease severity in several global studies [31,32], our analysis did not demonstrate a clear association between serotype and clinical or hematological indicators such as thrombocytopenia. This may reflect differences in study design, sample size, or the limited availability of detailed clinical data in our dataset. It is also possible that disease severity is influenced by multiple factors, including host immunity, secondary infections, and viral genotype, rather than serotype alone [33–35].

Climate-based studies in Pakistan show that rainfall, temperature, and urban population density play key roles in shaping dengue transmission, with Rawalpindi and Islamabad identified as transmission zones exhibiting a relatively long seasonal window from July to December (30). National data indicate that dengue outbreaks across major endemic cities consistently peak during the post-monsoon period despite year-to-year variation in case burden [10]. In contrast, Karachi’s warmer coastal climate and persistent humidity may support longer baseline vector survival, although dengue activity still typically peaks between September and November [22,36]. Overall, while Karachi may favor sustained transmission, Rawalpindi and Islamabad experience more pronounced seasonal surges, reflecting differences in transmission intensity versus duration across these environments.

The findings of this study have important public health implications for dengue prevention and control in Pakistan. The recurrent detection of dengue cases in Rawalpindi and Islamabad underscores the need for strengthened surveillance systems that integrate molecular diagnostics with epidemiological monitoring to detect shifts in circulating serotypes and anticipate outbreak dynamics. The predominance of cases from the twin cities (Rawalpindi and Islamabad) in our dataset reflects, in part, surveillance and referral bias. As the NIH in Islamabad functions as a national reference laboratory for dengue diagnostics, many hospitals from surrounding districts refer samples to this facility for confirmatory molecular testing. Furthermore, dengue serotyping capacity in Pakistan is currently largely centralized at NIH Islamabad, while most provincial public health reference laboratories do not routinely perform molecular serotyping. Consequently, the geographic distribution of samples in this study may not represent the true population-level burden of dengue across the country. In contrast, several major metropolitan regions, including Lahore and Karachi, maintain Provincial Public Health Reference Laboratories (PPHRLs) capable of performing local dengue diagnostics, meaning that many cases from these cities are managed locally and are therefore underrepresented in the present dataset. In addition, the study is based on passive laboratory-based surveillance, and detailed clinical data were available only for a subset of patients. Finally, genomic sequencing was not performed, limiting insights into viral lineage diversity and transmission dynamics. These limitations highlight the importance of interpreting the findings within the context of existing diagnostic infrastructure and surveillance systems in Pakistan.

This study’s major strength is the use of a large, multi-year dataset of molecularly confirmed dengue cases generated through a national reference laboratory, enabling robust assessment of temporal and geographic serotype dynamics. However, the study is based on passive, referral-based surveillance, which may not fully represent community-level dengue transmission. In addition, clinical and Hematological data were available for only a subset of patients, and information on primary versus secondary infections was not available. Differences in referral patterns and sample submission across years and districts may also have influenced the observed serotype distribution.

To improve the representativeness of dengue surveillance and strengthen national outbreak preparedness, future public health strategies should prioritize the expansion of decentralized molecular and genomic surveillance across major urban centers in Pakistan. While the NIH Pakistan currently serves as the national reference laboratory with advanced sequencing capacity, establishing routine molecular and genomic surveillance within PPHRLs would enable more comprehensive monitoring of viral evolution and geographic spread. An integrated surveillance network would facilitate early detection of emerging DENV lineages, including variants associated with increased transmission or virulence, as well as the introduction of new strains into urban transmission corridors. Early identification of such changes can provide critical warning signals for public health authorities, enabling timely vector control measures, improved clinical preparedness, and proactive outbreak response. Strengthening coordination between NIH Pakistan and provincial laboratories through standardized protocols and real-time data sharing will be essential for developing a more representative, responsive, and resilient national dengue surveillance system.

## Supporting information

S1 Table District-wise and year-wise distribution of dengue virus samples collected in Pakistan during 2021–2025. This table provides detailed breakdown of all NS1-positive serum samples received for serotyping, including district-wise distribution and yearly sample counts across the study period.
(XLSX)

S2 Table Anonymized dataset of qRT-PCR confirmed dengue virus serotyped samples (2021–2025). This table contains fully de-identified data of qRT-PCR confirmed dengue virus samples used for serotyping analysis during the study period.
(XLSX)
