# Supplementary material for: Multi-district molecular surveillance of dengue virus in Pakistan reveals serotype cycling and recurrent DENV-2 dominance, 2021–2025
Source: PLoS One. 2026 Jul 23;21(7):e0353487. doi: 10.1371/journal.pone.0353487 (PMC13395329; doi:10.1371/journal.pone.0353487)
Supplement: Attachment — Submitted filename: Response to Reviewers.docx [file pone.0353487.s004.docx]

**June 06, 2026**

**Response to Manuscript**

“**Multi-District Molecular Surveillance of Dengue Virus in Pakistan Reveals Serotype Cycling and Recurrent DENV-2 Dominance, 2021–2025”**

We thank the Editor and Reviewer for their valuable comments and constructive suggestions, which have helped improve the clarity and quality of the manuscript. The revised manuscript with tracked changes has been uploaded. All line numbers referenced in the rebuttal correspond to the simple line numbering in the tracked-changes version.

# Journal Requirements:

1. Please ensure that your manuscript meets PLOS ONE's style requirements, including those for file naming. The PLOS ONE style templates can be found at

**Response:** We have ensured correct naming.

2. We note that you have indicated that there are restrictions to data sharing for this study. PLOS only allows data to be available upon request if there are legal or ethical restrictions on sharing data publicly. For more information on unacceptable data access restrictions, please see <http://journals.plos.org/plosone/s/data-availability#loc-unacceptable-data-access-restrictions>.

Before we proceed with your manuscript, please address the following prompts:

a) If there are ethical or legal restrictions on sharing a de-identified data set, please explain them in detail (e.g., data contain potentially identifying or sensitive patient information, data are owned by a third-party organization, etc.) and who has imposed them (e.g., a Research Ethics Committee or Institutional Review Board, etc.). Please also provide contact information for a data access committee, ethics committee, or other institutional body to which data requests may be sent.

b) If there are no restrictions, please upload the minimal anonymized data set necessary to replicate your study findings to a stable, public repository and provide us with the relevant URLs, DOIs, or accession numbers. For a list of recommended repositories, please see

<https://journals.plos.org/plosone/s/recommended-repositories>. You also have the option of uploading the data as Supporting Information files, but we would recommend depositing data directly to a data repository if possible.

We will update your Data Availability statement on your behalf to reflect the information you provide.

3. Your ethics statement should only appear in the Methods section of your manuscript. If your ethics statement is written in any section besides the Methods, please delete it from any other section.

**Response:** We thank the editors for this comment. We confirm that there are no ethical or legal restrictions imposed by the Institutional Review Board (IRB) on sharing de-identified data from this study. We have now provided the minimal anonymized dataset necessary to replicate the study findings as **S2 Table**. All data included in this file have been fully de-identified and do not contain any information that could directly or indirectly identify participants. Researchers who wish to access additional data may submit a reasonable request via email to [fedsd@nih.org.pk](mailto:fedsd@nih.org.pk) or call at +92-51-9255237. Such requests will be reviewed by the National Institute of Health (NIH) and the IRB in accordance with applicable ethical and institutional policies. We will also update the manuscript’s Data Availability Statement accordingly. In addition, we confirm that the ethics statement is only present in the Methods section.

4. Please include a caption for figure 2.

The caption is included in manuscript.

5. We note that Figure 1 in your submission contain map images which may be copyrighted. All PLOS content is published under the Creative Commons Attribution License (CC BY 4.0), which means that the manuscript, images, and Supporting Information files will be freely available online, and any third party is permitted to access, download, copy, distribute, and use these materials in any way, even commercially, with proper attribution. For these reasons, we cannot publish previously copyrighted maps or satellite images created using proprietary data, such as Google software (Google Maps, Street View, and Earth). For more information, see our copyright guidelines: <http://journals.plos.org/plosone/s/licenses-and-copyright>.

 We require you to either (1) present written permission from the copyright holder to publish these figures specifically under the CC BY 4.0 license, or (2) remove the figures from your submission:

 1. You may seek permission from the original copyright holder of Figure 1 to publish the content specifically under the CC BY 4.0 license.

 We recommend that you contact the original copyright holder with the Content Permission Form (<http://journals.plos.org/plosone/s/file?id=7c09/content-permission-form.pdf>) and the following text:

“I request permission for the open-access journal PLOS ONE to publish XXX under the Creative Commons Attribution License (CCAL) CC BY 4.0 (<http://creativecommons.org/licenses/by/4.0/>). Please be aware that this license allows unrestricted use and distribution, even commercially, by third parties. Please reply and provide explicit written permission to publish XXX under a CC BY license and complete the attached form.”

 Please upload the completed Content Permission Form or other proof of granted permissions as an "Other" file with your submission.

In the figure caption of the copyrighted figure, please include the following text: “Reprinted from [ref] under a CC BY license, with permission from [name of publisher], original copyright [original copyright year].”

 2. If you are unable to obtain permission from the original copyright holder to publish these figures under the CC BY 4.0 license or if the copyright holder’s requirements are incompatible with the CC BY 4.0 license, please either i) remove the figure or ii) supply a replacement figure that complies with the CC BY 4.0 license. Please check copyright information on all replacement figures and update the figure caption with source information. If applicable, please specify in the figure caption text when a figure is similar but not identical to the original image and is therefore for illustrative purposes only.

The following resources for replacing copyrighted map figures may be helpful:

 USGS National Map Viewer (public domain): <http://viewer.nationalmap.gov/viewer/>

The Gateway to Astronaut Photography of Earth (public domain): <http://eol.jsc.nasa.gov/sseop/clickmap/>

Maps at the CIA (public domain): <https://www.cia.gov/library/publications/the-world-factbook/index.html> and <https://www.cia.gov/library/publications/cia-maps-publications/index.html>

NASA Earth Observatory (public domain): <http://earthobservatory.nasa.gov/>

Landsat: <http://landsat.visibleearth.nasa.gov/>

USGS EROS (Earth Resources Observatory and Science (EROS) Center) (public domain): [http://eros.usgs.gov/#](http://eros.usgs.gov/)

Natural Earth (public domain): <http://www.naturalearthdata.com/>

**Response:** We thank the reviewer for this important comment. The map used in Figure 1 was obtained from Wikimedia Commons (Pakistan districts SVG), which is explicitly released into the public domain by the copyright holder. Therefore, no additional permission is required, and the figure is fully compatible with the journal’s CC BY 4.0 licensing requirements. The figure caption has been updated accordingly. This is the link for map: ( https://commons.wikimedia.org/wiki/File:Map_of_Pakistan_%282018%29.svg)

6. If the reviewer comments include a recommendation to cite specific previously published works, please review and evaluate these publications to determine whether they are relevant and should be cited. There is no requirement to cite these works unless the editor has indicated otherwise.

**Response:** Noted.

**Specific comments**

1.To avoid confusion with NIH from other countries, it will be better to indicate that NIH in this manuscript is referring to National Institute of Health (NIH) Pakistan see line 21, 98, 269, 272, 285, & 293.

**Response:** We have added, NIH Pakistan for clarity at specified lines. (line number 14, 84, and others)

2.Line 71:  …twin cities:  Please clarify this as (Rawalpindi and Islamabad), since this term could have different meaning in different country.  For USA, twin cities mean  Minneapolis–Saint Paul.

**Response:** Added Rawalpindi and Islamabad as per the suggestion. (line number 263,283 and others)

3.Line 89,… between 2021 and 2024,… In the abstract and material and methods it is between 2021 and 2025. Which is the correct timeframe?

**Response:** The correct timeframe is 2021-2025. We have corrected. Line number 81

4.Line 112:  It will be helpful to the readers if the authors referred to Fig. 1 here.

**Response:** We thank the reviewer for this suggestion. We would like to clarify that the sample count mentioned in the Methods section (n = 1,644) refers to the total NS1-positive samples received for serotyping. In contrast, Figure 1 represents the geographic distribution of qRT-PCR–confirmed dengue cases (n = 1,162), which are presented in the Results section. Therefore, Fig. 1 is referenced in the Results rather than the Methods to avoid confusion between total received samples and confirmed cases used for serotype analysis.

5.Lin 112: Include a sentence indicate how many samples were excluded from analysis.

**Response:** We have included. Line: 102-104

6.Line 127:  Please add detail of qRT-PCR condition.,

**Response:** We have added cycling condition of qRT-PCR line 118-120

7.Line 137:  I counted 1171 samples from the description below, please clarify.

**Response:** We thank the editor for pointing this out. We have re-checked the data and confirm that the correct total is 1,162 qRT-PCR–confirmed cases. The error was due to a typographical issue and has now been corrected in the manuscript (Lines 135).

8.Line 155:  This should be Fig. 2 instead of Fig. 1. Also, change “Month wise” to “Monthly”

**Response:** We have changed month wise to monthly and corrected fig no. line number 156

**Reviewer #1:**

• In the abstract it is stated that “Gender-Age distribution showed majority of cases in males (64.5%)” but in the results it is stated that “Overall, dengue infections were more frequently observed in males (66%)”. One of these figures is incorrect and must be corrected consistently throughout.

**Response:** We thank the reviewer for carefully identifying this inconsistency. We confirm that the correct value is 66%. The abstract has been revised accordingly to ensure consistency throughout the manuscript. (line 23)

• In the abstract it is stated that DENV-3 is 0.08% “DENV-3 (0.08%)” but in the results it is stated that “DENV-3 remained sporadic, representing only 1.0% of cases in 2023 and was absent in other years”. These figures are contradictory. Add the number of cases along with percentages.

**Response:** We thank the reviewer for this comment. Both values are correct but were reported at different levels. DENV-3 represents 0.08% (1/1162) of total RT-qPCR–confirmed cases, and 1.0% in 2023 (single case). The manuscript has been revised to include both case numbers and percentages for consistency. (159-167)

• The title, abstract, and methods all state the study duration from 2021-2025 while the introduction states the study covers "2021 and 2024".

**Response:** The study period has been corrected in the Introduction to ensure consistency with the Title, Abstract, and Methods sections (2021–2025). (line 81)

• Both Figure 1 and Figure 2 labeled as “Fig 1”.

**Response:** We thank the reviewer for this comment. All figures have been already correctly labeled in the manuscript as Fig. 1, Fig. 2 and Fig.3, and the numbering is consistent throughout the text and figure captions. The formatting style (i.e., “Fig. 1”) follows the requirements of the PLOS journal guidelines.

• For 2025, the data is available till October, 2025 not December, 2025. Correct the study duration in methods and results section.

**Response:** We thank the reviewer for this observation. The study duration has been corrected in the Methods and Results sections to clearly indicate that the 2025 data are available up to October 2025. (line 85, 129)

• Of 1,644 NS1-positive samples, only 1,162 (70.7%) were confirmed positive by qRT-PCR. There is almost 30% discordance between NS1 antigen and qRT-PCR. The reason for this discrepancy and how it impacts the serotype distribution should be discussed.

**Response:** The observed difference is now mentioned at line 217-224 in discussion section.

• Clinical data available only for 604 patients but in Table 2, the total samples of the study (n=249 for DENV-1 and n=912 for DENV-2) used as denominators. This could inflate the sample size and impact the p-value. Re-run the analysis with only the samples for which the clinical data available.

**Response:** We thank the reviewer for this comment. We agree that using total qRT-PCR–confirmed cases as denominators was not appropriate given missing clinical data.We therefore re-ran the analyses using only cases with available clinical and hematological data (clinical: DENV-1 n=129, DENV-2 n=256; hematology: DENV-1 n=105, DENV-2 n=325). All percentages, chi-square tests, and p-values were recalculated, and updated results are shown in Table 3. (Line number 190-197)

• Serotype proportions are reported differently in the Abstract and Results. The author should provide a detailed table with numbers and percentages of serotype distribution over the years.

**Response:** We have added a detailed table (Table 1) showing the year-wise distribution of dengue virus serotypes, including absolute numbers and percentages. This table provides a clear breakdown which complements Fig. 2, thereby improving clarity and addressing the variation in serotype reporting between the Abstract and Results sections. We have also added details in text (line 159-167)

• Line 112 states that samples with "inappropriate conditions" or "incomplete clinical information" were excluded, but does not report how many samples were excluded for each reason.

**Response:** we have revised this line no 102-104.

• Figure 3 is not clear. Cannot determine the absolute numbers for the figure. Re-draw the figure with clarity.

**Response:** The figure is modified with absolute numbers shown.

**Reviewer #2:** I read with interest the manuscript by Umair et al. Multi-District Molecular Surveillance of Dengue Virus in Pakistan Reveals Serotype Cycling and Recurrent DENV-2 Dominance, 2021–2025. The dataset is clearly valuable, and the geographic scope is impressive. That said, I think the manuscript would benefit from some important clarifications before it is ready for publication. I have tried to be specific about what I would like to see addressed.

1. Abstract, Lines 35–36.
I appreciate the authors' transparency about referral bias, but the Abstract is not the right place for this disclaimer in its current form. As written, it reads like a footnote rather than useful information. I would suggest moving this point to the Discussion, where the authors can actually unpack what it means. For example, do referral patterns skew toward more severe cases, and if so, does that inflate the apparent dominance of DENV-2? If the authors prefer to keep some version of this in the Abstract, it needs to be more concrete—something like: "Because samples were referral-based, DENV-2 may be overrepresented, given this serotype infection is often associated with severe dengue fever and may prompt patients to visit hospitals or healthcare more than DF patients infected with DENV1 or DENV3.

**Response:** We thank the reviewer for this valuable suggestion. We agree that the statement regarding referral-based sampling was better suited to the Discussion than the Abstract. Accordingly, the sentence was removed from the Abstract, and the Discussion was revised as per suggestion. (line 286-290)

2. Lines 53–55.
Secondary DENV infection is a risk factor for DHF. Given the well-established role of antibody-dependent enhancement and the fact that DENV-2 dominance with sporadic DENV-1 circulation in late 2022 and 2023, and rare DENV3 infection occurs in some districts. However, this doesn’t mean that DENV1 and DENV3 are rare in the communities because the peripheral healthcare-based or community-wide genomics surveillance had not been done to capture mild or subclinical cases, most likely with infection with DENV3 or DENV1. A brief paragraph framing this would help. If the authors do not have primary/secondary infection data for these samples, that is completely understandable, but it should be stated explicitly as a limitation rather than omitted entirely.

**Response:** We thank the reviewer for this important suggestion. A paragraph has been added in the Discussion to clarify that the observed serotype distribution is based on passive hospital-based surveillance and may not reflect true community-level circulation. We also acknowledge that data on primary and secondary infections were not available in this study, and this has been explicitly stated as a limitation. Line (286-298, 301-306)

3. Lines 59–60 and Line 195. Minor point, but "dengue virus" is defined at Line 41. Line 59 expanded again. On the other hand, line 195 still has "Dengue virus" written out in full. DENV was already expanded in line 41 and throughout thereafter, DENV should have been used. Same for DHF.

The manuscript has been revised to ensure consistency in terminology.

4. Lines 120–121: Missing detail on the kit.
Could the authors add the city for Qiagen? I believe it is Hilden, but the manuscript should specify for reproducibility.

**Response:** We have added city. Line no 112

5. Figure 1: The pie charts representing the different representations of different DENV serotypes across the Pakistan districts
I found the current visualization difficult to interpret in a meaningful way. All pies are the same size, so a district with 500 samples looks identical to one with 20. That makes it impossible to judge whether the serotype proportions reflect genuine epidemiology or simply small-sample noise. I would strongly encourage the authors to scale the pie areas to the sample size and to report absolute counts rather than percentages. Additionally, it would help to readers if authors label each district with sampling years, since Figure 1 currently gives the impression of a uniform temporal snapshot when the text makes clear that sampling years differ by district.

**Response:** We thank the reviewer for this suggestion. We would like to mention that Figure 1 already presents the absolute number of cases (n) and the corresponding serotype percentages within each pie chart. In addition, district-level case counts are represented by the existing green color gradient on the map. We explored scaling pie-chart sizes according to sample numbers; however, due to the large variation in case counts across districts, this resulted in a more complex figure and reduced the visibility of serotype distributions in districts with smaller sample sizes. Therefore, we retained uniform pie sizes while displaying the absolute counts and percentages. To further improve clarity, we have revised the figure caption to emphasize these features and have also included S1 Table.

6. Relationship to Jama et al. (2025).
I noticed a very recent paper by some of the same authors in Infection, Genetics and Evolution covering dengue in Pakistan during 2024. I am not suggesting anything improper, but as a reader, I genuinely could not tell whether the two papers analyze overlapping datasets, completely separate datasets, or some combination. For the readers, as they may encounter it as I did, it would be good if the authors clarify: (a) whether any sequences or data in this manuscript were published in the Jama et al. paper; (b) whether the geographic or temporal scope differs; and (c) whether the conclusions are consistent between the two studies. This kind of up-front transparency helps readers (and reviewers) understand what is genuinely new here.

**Response:** We would like to clarify that the study by Jamal et al. (2025) focused on genomic characterization and sequencing of dengue virus from the 2024 outbreak in Pakistan, whereas the present study analyzes a broader 2021–2025 dataset to assess temporal and geographic serotype dynamics using qRT-PCR–based surveillance. Although both studies include samples processed at NIH, the datasets differ in scope and analytical focus. There is partial overlap in institutional sample sources for 2024; however, the sequencing data and genomic analyses reported in Jamal et al. (2025) are not included in this manuscript. The conclusions of both studies are consistent regarding DENV-2 predominance, while the present study extends these findings to a multi-year epidemiological framework. We have added line in discussion [Line 253-255]

7. The section for strengths and limitations is missing
I found no section on the strengths and limitations in the manuscript. I believe the section is important for a surveillance study that relies on convenience sampling. I would like to read how the authors address this. Specifically, how might referral patterns distort the serotype map? What clinical variables are missing, and how does that constrain interpretation? Are the apparent shifts in serotype dominance real epidemiological turnover, or could they reflect uneven sampling intensity across years and districts?

**Response:** We thank the reviewer for this valuable suggestion. A dedicated Strengths and Limitations section has been added to the Discussion as per suggestion. Line: 299-306
