# Supplementary material for: Multi-district molecular surveillance of dengue virus in Pakistan reveals serotype cycling and recurrent DENV-2 dominance, 2021–2025
Source: PLoS One. 2026 Jul 23;21(7):e0353487. doi: 10.1371/journal.pone.0353487 (PMC13395329; doi:10.1371/journal.pone.0353487)
Supplement: Peer review history

# Peer review: Decision Letter 0

**DOI:** 10.1371/journal.pone.0353487.r001

**Authors:** Baochuan Lin

13 May 2026

PONE-D-26-13662

Multi-District Molecular Surveillance of Dengue Virus in Pakistan Reveals Serotype Cycling and Recurrent DENV-2 Dominance, 2021–2025

PLOS One

Dear Dr. Umair,

Thank you for submitting your manuscript to PLOS ONE. After careful consideration, we feel that it has merit but does not fully meet PLOS ONE’s publication criteria as it currently stands. Therefore, we invite you to submit a revised version of the manuscript that addresses the points raised during the review process.

Please submit your revised manuscript by Jun 27 2026 11:59PM. If you will need more time than this to complete your revisions, please reply to this message or contact the journal office at plosone@plos.org. When you're ready to submit your revision, log on to https://www.editorialmanager.com/pone/ and select the 'Submissions Needing Revision' folder to locate your manuscript file.

Please include the following items when submitting your revised manuscript:

- A letter that responds to each point raised by the academic editor and reviewer(s). You should upload this letter as a separate file labeled 'Response to Reviewers'.
- A marked-up copy of your manuscript that highlights changes made to the original version. You should upload this as a separate file labeled 'Revised Manuscript with Track Changes'.
- An unmarked version of your revised paper without tracked changes. You should upload this as a separate file labeled 'Manuscript'.

If you would like to make changes to your financial disclosure, please include your updated statement in your cover letter. Guidelines for resubmitting your figure files are available below the reviewer comments at the end of this letter.

If applicable, we recommend that you deposit your laboratory protocols in protocols.io to enhance the reproducibility of your results. Protocols.io assigns your protocol its own identifier (DOI) so that it can be cited independently in the future. For instructions see: https://journals.plos.org/plosone/s/submission-guidelines#loc-laboratory-protocols. Additionally, PLOS ONE offers an option for publishing peer-reviewed Lab Protocol articles, which describe protocols hosted on protocols.io. Read more information on sharing protocols at https://plos.org/protocols?utm_medium=editorial-email&utm_source=authorletters&utm_campaign=protocols.

As the corresponding author, your ORCID iD is verified in the submission system and will appear in the published article. PLOS supports the use of ORCID, and we encourage all coauthors to register for an ORCID iD and use it as well. Please encourage your coauthors to verify their ORCID iD within the submission system before final acceptance, as unverified ORCID iDs will not appear in the published article. *Only*  the individual author can complete the verification step; PLOS staff *cannot*  verify ORCID iDs on behalf of authors.

We look forward to receiving your revised manuscript.

Kind regards,

Baochuan Lin, Ph.D.

Academic Editor

PLOS One

Journal Requirements:

When submitting your revision, we need you to address these additional requirements.

1.Please ensure that your manuscript meets PLOS ONE's style requirements, including those for file naming. The PLOS ONE style templates can be found at

https://journals.plos.org/plosone/s/file?id=wjVg/PLOSOne_formatting_sample_main_body.pdf and

https://journals.plos.org/plosone/s/file?id=ba62/PLOSOne_formatting_sample_title_authors_affiliations.pdf

2. We note that you have indicated that there are restrictions to data sharing for this study. PLOS only allows data to be available upon request if there are legal or ethical restrictions on sharing data publicly. For more information on unacceptable data access restrictions, please see http://journals.plos.org/plosone/s/data-availability#loc-unacceptable-data-access-restrictions.

Before we proceed with your manuscript, please address the following prompts:

a) If there are ethical or legal restrictions on sharing a de-identified data set, please explain them in detail (e.g., data contain potentially identifying or sensitive patient information, data are owned by a third-party organization, etc.) and who has imposed them (e.g., a Research Ethics Committee or Institutional Review Board, etc.). Please also provide contact information for a data access committee, ethics committee, or other institutional body to which data requests may be sent.

b) If there are no restrictions, please upload the minimal anonymized data set necessary to replicate your study findings to a stable, public repository and provide us with the relevant URLs, DOIs, or accession numbers. For a list of recommended repositories, please see

https://journals.plos.org/plosone/s/recommended-repositories. You also have the option of uploading the data as Supporting Information files, but we would recommend depositing data directly to a data repository if possible.

We will update your Data Availability statement on your behalf to reflect the information you provide.

3. Your ethics statement should only appear in the Methods section of your manuscript. If your ethics statement is written in any section besides the Methods, please delete it from any other section.

4. Please include a caption for figure 2.

5. We note that Figure 1 in your submission contain map images which may be copyrighted. All PLOS content is published under the Creative Commons Attribution License (CC BY 4.0), which means that the manuscript, images, and Supporting Information files will be freely available online, and any third party is permitted to access, download, copy, distribute, and use these materials in any way, even commercially, with proper attribution. For these reasons, we cannot publish previously copyrighted maps or satellite images created using proprietary data, such as Google software (Google Maps, Street View, and Earth). For more information, see our copyright guidelines: http://journals.plos.org/plosone/s/licenses-and-copyright.

We require you to either (1) present written permission from the copyright holder to publish these figures specifically under the CC BY 4.0 license, or (2) remove the figures from your submission:

1. You may seek permission from the original copyright holder of Figure 1 to publish the content specifically under the CC BY 4.0 license.

We recommend that you contact the original copyright holder with the Content Permission Form (http://journals.plos.org/plosone/s/file?id=7c09/content-permission-form.pdf) and the following text:

“I request permission for the open-access journal PLOS ONE to publish XXX under the Creative Commons Attribution License (CCAL) CC BY 4.0 (http://creativecommons.org/licenses/by/4.0/). Please be aware that this license allows unrestricted use and distribution, even commercially, by third parties. Please reply and provide explicit written permission to publish XXX under a CC BY license and complete the attached form.”

Please upload the completed Content Permission Form or other proof of granted permissions as an "Other" file with your submission.

In the figure caption of the copyrighted figure, please include the following text: “Reprinted from [ref] under a CC BY license, with permission from [name of publisher], original copyright [original copyright year].”

2. If you are unable to obtain permission from the original copyright holder to publish these figures under the CC BY 4.0 license or if the copyright holder’s requirements are incompatible with the CC BY 4.0 license, please either i) remove the figure or ii) supply a replacement figure that complies with the CC BY 4.0 license. Please check copyright information on all replacement figures and update the figure caption with source information. If applicable, please specify in the figure caption text when a figure is similar but not identical to the original image and is therefore for illustrative purposes only.

The following resources for replacing copyrighted map figures may be helpful:

USGS National Map Viewer (public domain): http://viewer.nationalmap.gov/viewer/

The Gateway to Astronaut Photography of Earth (public domain): http://eol.jsc.nasa.gov/sseop/clickmap/

Maps at the CIA (public domain): https://www.cia.gov/library/publications/the-world-factbook/index.html and https://www.cia.gov/library/publications/cia-maps-publications/index.html

NASA Earth Observatory (public domain): http://earthobservatory.nasa.gov/

Landsat: http://landsat.visibleearth.nasa.gov/

USGS EROS (Earth Resources Observatory and Science (EROS) Center) (public domain): http://eros.usgs.gov/#

Natural Earth (public domain): http://www.naturalearthdata.com/

6. If the reviewer comments include a recommendation to cite specific previously published works, please review and evaluate these publications to determine whether they are relevant and should be cited. There is no requirement to cite these works unless the editor has indicated otherwise.

Additional Editor Comments:

I have received the reviews of your manuscript. While your paper addresses an interesting question, the reviewers stated several concerns about your study and did not recommend publication in its present form.  Please see reviewers’ insightful comments below. These comments need to be addressed carefully.  In addition, there are a few confusing points in the manuscript that need to be clarified, please see specific comments below.

Specific comments

1.To avoid confusion with NIH from other countries, it will be better to indicate that NIH in this manuscript is referring to National Institute of Health (NIH) Pakistan see line 21, 98, 269, 272, 285, & 293.

2.Line 71:  …twin cities:  Please clarify this as (Rawalpindi and Islamabad), since this term could have different meaning in different country.  For USA, twin cities mean  Minneapolis–Saint Paul.

3.Line 89,… between 2021 and 2024,… In the abstract and material and methods it is between 2021 and 2025. Which is the correct timeframe?

4.Line 112:  It will be helpful to the readers if the authors referred to Fig. 1 here.

5.Lin 112: Include a sentence indicate how many samples were excluded from analysis.

6.Line 127:  Please add detail of qRT-PCR condition.

7.Line 137:  I counted 1171 samples from the description below, please clarify.

8.Line 155:  This should be Fig. 2 instead of Fig. 1. Also, change “Month wise” to “Monthly”

[Note: HTML markup is below. Please do not edit.]

Reviewers' comments:

Reviewer's Responses to Questions

**Comments to the Author**

1. Is the manuscript technically sound, and do the data support the conclusions?

The manuscript must describe a technically sound piece of scientific research with data that supports the conclusions. Experiments must have been conducted rigorously, with appropriate controls, replication, and sample sizes. The conclusions must be drawn appropriately based on the data presented.

Reviewer #1: Yes

Reviewer #2: Yes

**********

2. Has the statistical analysis been performed appropriately and rigorously?

Reviewer #1: No

Reviewer #2: Yes

**********

3. Have the authors made all data underlying the findings in their manuscript fully available?

The PLOS Data policy requires authors to make all data underlying the findings described in their manuscript fully available without restriction, with rare exception (please refer to the Data Availability Statement in the manuscript PDF file). The data should be provided as part of the manuscript or its supporting information, or deposited to a public repository. For example, in addition to summary statistics, the data points behind means, medians and variance measures should be available. If there are restrictions on publicly sharing data—e.g. participant privacy or use of data from a third party—those must be specified.

Reviewer #1: Yes

Reviewer #2: Yes

**********

4. Is the manuscript presented in an intelligible fashion and written in standard English?

PLOS ONE does not copyedit accepted manuscripts, so the language in submitted articles must be clear, correct, and unambiguous. Any typographical or grammatical errors should be corrected at revision, so please note any specific errors here.

Reviewer #1: Yes

Reviewer #2: Yes

**********

5. Review Comments to the Author

Please use the space provided to explain your answers to the questions above. You may also include additional comments for the author, including concerns about dual publication, research ethics, or publication ethics. (Please upload your review as an attachment if it exceeds 20,000 characters)

Reviewer #1: • In the abstract it is stated that “Gender-Age distribution showed majority of cases in males (64.5%)” but in the results it is stated that “Overall, dengue infections were more frequently observed in males (66%)”. One of these figures is incorrect and must be corrected consistently throughout.

• In the abstract it is stated that DENV-3 is 0.08% “DENV-3 (0.08%)” but in the results it is stated that “DENV-3 remained sporadic, representing only 1.0% of cases in 2023 and was absent in other years”. These figures are contradictory. Add the number of cases along with percentages.

• The title, abstract, and methods all state the study duration from 2021-2025 while the introduction states the study covers "2021 and 2024".

• Both Figure 1 and Figure 2 labeled as “Fig 1”.

• For 2025, the data is available till October, 2025 not December, 2025. Correct the study duration in methods and results section.

• Of 1,644 NS1-positive samples, only 1,162 (70.7%) were confirmed positive by qRT-PCR. There is almost 30% discordance between NS1 antigen and qRT-PCR. The reason for this discrepancy and how it impacts the serotype distribution should be discussed.

• Clinical data available only for 604 patients but in Table 2, the total samples of the study (n=249 for DENV-1 and n=912 for DENV-2) used as denominators. This could inflate the sample size and impact the p-value. Re-run the analysis with only the samples for which the clinical data available.

• Serotype proportions are reported differently in the Abstract and Results. The author should provide a detailed table with numbers and percentages of serotype distribution over the years.

• Line 112 states that samples with "inappropriate conditions" or "incomplete clinical information" were excluded, but does not report how many samples were excluded for each reason.

• Figure 3 is not clear. Cannot determine the absolute numbers for the figure. Re-draw the figure with clarity.

Reviewer #2: I read with interest the manuscript by Umair et al. Multi-District Molecular Surveillance of Dengue Virus in Pakistan Reveals Serotype Cycling and Recurrent DENV-2 Dominance, 2021–2025. The dataset is clearly valuable, and the geographic scope is impressive. That said, I think the manuscript would benefit from some important clarifications before it is ready for publication. I have tried to be specific about what I would like to see addressed.

1. Abstract, Lines 35–36.

I appreciate the authors' transparency about referral bias, but the Abstract is not the right place for this disclaimer in its current form. As written, it reads like a footnote rather than useful information. I would suggest moving this point to the Discussion, where the authors can actually unpack what it means. For example, do referral patterns skew toward more severe cases, and if so, does that inflate the apparent dominance of DENV-2? If the authors prefer to keep some version of this in the Abstract, it needs to be more concrete—something like: "Because samples were referral-based, DENV-2 may be overrepresented, given this serotype infection is often associated with severe dengue fever and may prompt patients to visit hospitals or healthcare more than DF patients infected with DENV1 or DENV3.

2. Lines 53–55.

Secondary DENV infection is a risk factor for DHF. Given the well-established role of antibody-dependent enhancement and the fact that DENV-2 dominance with sporadic DENV-1 circulation in late 2022 and 2023, and rare DENV3 infection occurs in some districts. However, this doesn’t mean that DENV1 and DENV3 are rare in the communities because the peripheral healthcare-based or community-wide genomics surveillance had not been done to capture mild or subclinical cases, most likely with infection with DENV3 or DENV1. A brief paragraph framing this would help. If the authors do not have primary/secondary infection data for these samples, that is completely understandable, but it should be stated explicitly as a limitation rather than omitted entirely.

3. Lines 59–60 and Line 195. Minor point, but "dengue virus" is defined at Line 41. Line 59 expanded again. On the other hand, line 195 still has "Dengue virus" written out in full. DENV was already expanded in line 41 and throughout thereafter, DENV should have been used. Same for DHF.

4. Lines 120–121: Missing detail on the kit.

Could the authors add the city for Qiagen? I believe it is Hilden, but the manuscript should specify for reproducibility.

5. Figure 1: The pie charts representing the different representations of different DENV serotypes across the Pakistan districts

I found the current visualization difficult to interpret in a meaningful way. All pies are the same size, so a district with 500 samples looks identical to one with 20. That makes it impossible to judge whether the serotype proportions reflect genuine epidemiology or simply small-sample noise. I would strongly encourage the authors to scale the pie areas to the sample size and to report absolute counts rather than percentages. Additionally, it would help to readers if authors label each district with sampling years, since Figure 1 currently gives the impression of a uniform temporal snapshot when the text makes clear that sampling years differ by district.

6. Relationship to Jama et al. (2025).

I noticed a very recent paper by some of the same authors in Infection, Genetics and Evolution covering dengue in Pakistan during 2024. I am not suggesting anything improper, but as a reader, I genuinely could not tell whether the two papers analyze overlapping datasets, completely separate datasets, or some combination. For the readers, as they may encounter it as I did, it would be good if the authors clarify: (a) whether any sequences or data in this manuscript were published in the Jama et al. paper; (b) whether the geographic or temporal scope differs; and (c) whether the conclusions are consistent between the two studies. This kind of up-front transparency helps readers (and reviewers) understand what is genuinely new here.

7. The section for strengths and limitations is missing

I found no section on the strengths and limitations in the manuscript. I believe the section is important for a surveillance study that relies on convenience sampling. I would like to read how the authors address this. Specifically, how might referral patterns distort the serotype map? What clinical variables are missing, and how does that constrain interpretation? Are the apparent shifts in serotype dominance real epidemiological turnover, or could they reflect uneven sampling intensity across years and districts?

**********

6. PLOS authors have the option to publish the peer review history of their article (what does this mean?). If published, this will include your full peer review and any attached files.

If you choose “no”, your identity will remain anonymous but your review may still be made public.

**Do you want your identity to be public for this peer review?**  For information about this choice, including consent withdrawal, please see our Privacy Policy.

Reviewer #1: No

Reviewer #2: **Yes:** Tesfaye Gelanew

**********

[NOTE: If reviewer comments were submitted as an attachment file, they will be attached to this email and accessible via the submission site. Please log into your account, locate the manuscript record, and check for the action link "View Attachments". If this link does not appear, there are no attachment files.]

To ensure your figures meet our technical requirements, please review our figure guidelines: https://journals.plos.org/plosone/s/figures

You may also use PLOS’s free figure tool, NAAS, to help you prepare publication quality figures: https://journals.plos.org/plosone/s/figures#loc-tools-for-figure-preparation.

NAAS will assess whether your figures meet our technical requirements by comparing each figure against our figure specifications.

# Author comment: Author response to Decision Letter 1

**DOI:** 10.1371/journal.pone.0353487.r002

10 Jun 2026

June 06, 2026

Response to Manuscript

“Multi-District Molecular Surveillance of Dengue Virus in Pakistan Reveals Serotype Cycling and Recurrent DENV-2 Dominance, 2021–2025”

We thank the Editor and Reviewer for their valuable comments and constructive suggestions, which have helped improve the clarity and quality of the manuscript. The revised manuscript with tracked changes has been uploaded. All line numbers referenced in the rebuttal correspond to the simple line numbering in the tracked-changes version.

Journal Requirements:

1. Please ensure that your manuscript meets PLOS ONE's style requirements, including those for file naming. The PLOS ONE style templates can be found at

Response: We have ensured correct naming.

2. We note that you have indicated that there are restrictions to data sharing for this study. PLOS only allows data to be available upon request if there are legal or ethical restrictions on sharing data publicly. For more information on unacceptable data access restrictions, please see http://journals.plos.org/plosone/s/data-availability#loc-unacceptable-data-access-restrictions.

Before we proceed with your manuscript, please address the following prompts:

a) If there are ethical or legal restrictions on sharing a de-identified data set, please explain them in detail (e.g., data contain potentially identifying or sensitive patient information, data are owned by a third-party organization, etc.) and who has imposed them (e.g., a Research Ethics Committee or Institutional Review Board, etc.). Please also provide contact information for a data access committee, ethics committee, or other institutional body to which data requests may be sent.

b) If there are no restrictions, please upload the minimal anonymized data set necessary to replicate your study findings to a stable, public repository and provide us with the relevant URLs, DOIs, or accession numbers. For a list of recommended repositories, please see

https://journals.plos.org/plosone/s/recommended-repositories. You also have the option of uploading the data as Supporting Information files, but we would recommend depositing data directly to a data repository if possible.

We will update your Data Availability statement on your behalf to reflect the information you provide.

3. Your ethics statement should only appear in the Methods section of your manuscript. If your ethics statement is written in any section besides the Methods, please delete it from any other section.

Response: We thank the editors for this comment. We confirm that there are no ethical or legal restrictions imposed by the Institutional Review Board (IRB) on sharing de-identified data from this study. We have now provided the minimal anonymized dataset necessary to replicate the study findings as S2 Table. All data included in this file have been fully de-identified and do not contain any information that could directly or indirectly identify participants. Researchers who wish to access additional data may submit a reasonable request via email to fedsd@nih.org.pk or call at +92-51-9255237. Such requests will be reviewed by the National Institute of Health (NIH) and the IRB in accordance with applicable ethical and institutional policies. We will also update the manuscript’s Data Availability Statement accordingly. In addition, we confirm that the ethics statement is only present in the Methods section.

4. Please include a caption for figure 2.

The caption is included in manuscript.

5. We note that Figure 1 in your submission contain map images which may be copyrighted. All PLOS content is published under the Creative Commons Attribution License (CC BY 4.0), which means that the manuscript, images, and Supporting Information files will be freely available online, and any third party is permitted to access, download, copy, distribute, and use these materials in any way, even commercially, with proper attribution. For these reasons, we cannot publish previously copyrighted maps or satellite images created using proprietary data, such as Google software (Google Maps, Street View, and Earth). For more information, see our copyright guidelines: http://journals.plos.org/plosone/s/licenses-and-copyright.

We require you to either (1) present written permission from the copyright holder to publish these figures specifically under the CC BY 4.0 license, or (2) remove the figures from your submission:

1. You may seek permission from the original copyright holder of Figure 1 to publish the content specifically under the CC BY 4.0 license.

We recommend that you contact the original copyright holder with the Content Permission Form (http://journals.plos.org/plosone/s/file?id=7c09/content-permission-form.pdf) and the following text:

“I request permission for the open-access journal PLOS ONE to publish XXX under the Creative Commons Attribution License (CCAL) CC BY 4.0 (http://creativecommons.org/licenses/by/4.0/). Please be aware that this license allows unrestricted use and distribution, even commercially, by third parties. Please reply and provide explicit written permission to publish XXX under a CC BY license and complete the attached form.”

Please upload the completed Content Permission Form or other proof of granted permissions as an "Other" file with your submission.

In the figure caption of the copyrighted figure, please include the following text: “Reprinted from [ref] under a CC BY license, with permission from [name of publisher], original copyright [original copyright year].”

2. If you are unable to obtain permission from the original copyright holder to publish these figures under the CC BY 4.0 license or if the copyright holder’s requirements are incompatible with the CC BY 4.0 license, please either i) remove the figure or ii) supply a replacement figure that complies with the CC BY 4.0 license. Please check copyright information on all replacement figures and update the figure caption with source information. If applicable, please specify in the figure caption text when a figure is similar but not identical to the original image and is therefore for illustrative purposes only.

The following resources for replacing copyrighted map figures may be helpful:

USGS National Map Viewer (public domain): http://viewer.nationalmap.gov/viewer/

The Gateway to Astronaut Photography of Earth (public domain): http://eol.jsc.nasa.gov/sseop/clickmap/

Maps at the CIA (public domain): https://www.cia.gov/library/publications/the-world-factbook/index.html and https://www.cia.gov/library/publications/cia-maps-publications/index.html

NASA Earth Observatory (public domain): http://earthobservatory.nasa.gov/

Landsat: http://landsat.visibleearth.nasa.gov/

USGS EROS (Earth Resources Observatory and Science (EROS) Center) (public domain): http://eros.usgs.gov/#

Natural Earth (public domain): http://www.naturalearthdata.com/

Response: We thank the reviewer for this important comment. The map used in Figure 1 was obtained from Wikimedia Commons (Pakistan districts SVG), which is explicitly released into the public domain by the copyright holder. Therefore, no additional permission is required, and the figure is fully compatible with the journal’s CC BY 4.0 licensing requirements. The figure caption has been updated accordingly. This is the link for map: ( https://commons.wikimedia.org/wiki/File:Map_of_Pakistan_%282018%29.svg)

6. If the reviewer comments include a recommendation to cite specific previously published works, please review and evaluate these publications to determine whether they are relevant and should be cited. There is no requirement to cite these works unless the editor has indicated otherwise.

Response: Noted.

Specific comments

1.To avoid confusion with NIH from other countries, it will be better to indicate that NIH in this manuscript is referring to National Institute of Health (NIH) Pakistan see line 21, 98, 269, 272, 285, & 293.

Response: We have added, NIH Pakistan for clarity at specified lines. (line number 14, 84, and others)

2.Line 71: …twin cities: Please clarify this as (Rawalpindi and Islamabad), since this term could have different meaning in different country. For USA, twin cities mean Minneapolis–Saint Paul.

Response: Added Rawalpindi and Islamabad as per the suggestion. (line number 263,283 and others)

3.Line 89,… between 2021 and 2024,… In the abstract and material and methods it is between 2021 and 2025. Which is the correct timeframe?

Response: The correct timeframe is 2021-2025. We have corrected. Line number 81

4.Line 112: It will be helpful to the readers if the authors referred to Fig. 1 here.

Response: We thank the reviewer for this suggestion. We would like to clarify that the sample count mentioned in the Methods section (n = 1,644) refers to the total NS1-positive samples received for serotyping. In contrast, Figure 1 represents the geographic distribution of qRT-PCR–confirmed dengue cases (n = 1,162), which are presented in the Results section. Therefore, Fig. 1 is referenced in the Results rather than the Methods to avoid confusion between total received samples and confirmed cases used for serotype analysis.

5.Lin 112: Include a sentence indicate how many samples were excluded from analysis.

Response: We have included. Line: 102-104

6.Line 127: Please add detail of qRT-PCR condition.,

Response: We have added cycling condition of qRT-PCR line 118-120

7.Line 137: I counted 1171 samples from the description below, please clarify.

Response: We thank the editor for pointing this out. We have re-checked the data and confirm that the correct total is 1,162 qRT-PCR–confirmed cases. The error was due to a typographical issue and has now been corrected in the manuscript (Lines 135).

8.Line 155: This should be Fig. 2 instead of Fig. 1. Also, change “Month wise” to “Monthly”

Response: We have changed month wise to monthly and corrected fig no. line number 156

Reviewer #1:

• In the abstract it is stated that “Gender-Age distribution showed majority of cases in males (64.5%)” but in the results it is stated that “Overall, dengue infections were more frequently observed in males (66%)”. One of these figures is incorrect and must be corrected consistently throughout.

Response: We thank the reviewer for carefully identifying this inconsistency. We confirm that the correct value is 66%. The abstract has been revised accordingly to ensure consistency throughout the manuscript. (line 23)

• In the abstract it is stated that DENV-3 is 0.08% “DENV-3 (0.08%)” but in the results it is stated that “DENV-3 remained sporadic, representing only 1.0% of cases in 2023 and was absent in other years”. These figures are contradictory. Add the number of cases along with percentages.

Response: We thank the reviewer for this comment. Both values are correct but were reported at different levels. DENV-3 represents 0.08% (1/1162) of total RT-qPCR–confirmed cases, and 1.0% in 2023 (single case). The manuscript has been revised to include both case numbers and percentages for consistency. (159-167)

• The title, abstract, and methods all state the study duration from 2021-2025 while the introduction states the study covers "2021 and 2024".

Response: The study period has been corrected in the Introduction to ensure consistency with the Title, Abstract, and Methods sections (2021–2025). (line 81)

• Both Figure 1 and Figure 2 labeled as “Fig 1”.

Response: We thank the reviewer for this comment. All figures have been already correctly labeled in the manuscript as Fig. 1, Fig. 2 and Fig.3, and the numbering is consistent throughout the text and figure captions. The formatting style (i.e., “Fig. 1”) follows the requirements of the PLOS journal guidelines.

• For 2025, the data is available till October, 2025 not December, 2025. Correct the study duration in methods and results section.

Response: We thank the reviewer for this observation. The study duration has been corrected in the Methods and Results sections to clearly indicate that the 2025 data are available up to October 2025. (line 85, 129)

• Of 1,644 NS1-positive samples, only 1,162 (70.7%) were confirmed positive by qRT-PCR. There is almost 30% discordance between NS1 antigen and qRT-PCR. The reason for this discrepancy and how it impacts the serotype distribution should be discussed.

Response: The observed difference is now mentioned at line 217-224 in discussion section.

• Clinical data available only for 604 patients but in Table 2, the total samples of the study (n=249 for DENV-1 and n=912 for DENV-2) used as denominators. This could inflate the sample size and impact the p-value. Re-run the analysis with only the samples for which the clinical data available.

Response: We thank the reviewer for this comment. We agree that using total qRT-PCR–confirmed cases as denominators was not appropriate given missing clinical data.We therefore re-ran the analyses using only cases with available clinical and hematological data (clinical: DENV-1 n=129, DENV-2 n=256; hematology: DENV-1 n=105, DENV-2 n=325). All percentages, chi-square tests, and p-values were recalculated, and updated results are shown in Table 3. (Line number 190-197)

• Serotype proportions are reported differently in the Abstract and Results. The author should provide a detailed table with numbers and percentages of serotype distribution over the years.

Response: We have added a detailed table (Table 1) showing the year-wise distribution of dengue virus serotypes, including absolute numbers and percentages. This table provides a clear breakdown which complements Fig. 2, thereby improving clarity and addressing the variation in serotype reporting between the Abstract and Results sections. We have also added details in text (line 159-167)

• Line 112 states that samples with "inappropriate conditions" or "incomplete clinical information" were excluded, but does not report how many samples were excluded for each reason.

Response: we have revised this line no 102-104.

• Figure 3 is not clear. Cannot determine the absolute numbers for the figure. Re-draw the figure with clarity.

Response: The figure is modified with absolute numbers shown.

Reviewer #2: I read with interest the manuscript by Umair et al. Multi-District Molecular Surveillance of Dengue Virus in Pakistan Reveals Serotype Cycling and Recurrent DENV-2 Dominance, 2021–2025. The dataset is clearly valuable, and the geographic scope is impressive. That said, I think the manuscript would benefit from some important clarifications before it is ready for publication. I have tried to be specific about what I would like to see addressed.

1. Abstract, Lines 35–36.

I appreciate the authors' transparency about referral bias, but the Abstract is not the right place for this disclaimer in its current form. As written, it reads like a footnote rather than useful information. I would suggest moving this point to the Discussion, where the authors can actually unpack what it means. For example, do referral patterns skew toward more severe cases, and if so, does that inflate the apparent dominance of DENV-2? If the authors prefer to keep some version of this in the Abstract, it needs to be more concrete—something like: "Because samples were referral-based, DENV-2 may be overrepresented, given this serotype infection is often associated with severe dengue fever and may prompt patients to visit hospitals or healthcare more than DF patients infected with DENV1 or DENV3.

Response: We thank the reviewer for this valuable suggestion. We agree that the statement regarding referral-based sampling was better suited to the Discussion than the Abstract. Accordingly, the sentence was removed from the Abstract, and the Discussion was revised as per suggestion. (line 286-290)

2. Lines 53–55.

Secondary DENV infection is a risk factor for DHF. Given the well-established role of antibody-dependent enhancement and the fact that DENV-2 dominance with sporadic DENV-1 circulation in late 2022 and 2023, and rare DENV3 infection occurs in some districts. However, this doesn’t mean that DENV1 and DENV3 are rare in the communities because the peripheral healthcare-based or community-wide genomics surveillance had not been done to capture mild or subclinical cases, most likely with infection with DENV3 or DENV1. A b

Attachment Submitted filename: Response to Reviewers.docx

# Peer review: Decision Letter 1

**DOI:** 10.1371/journal.pone.0353487.r003

**Authors:** Baochuan Lin

25 Jun 2026

Multi-district molecular surveillance of dengue virus in Pakistan reveals serotype cycling and recurrent DENV-2 dominance, 2021–2025

PONE-D-26-13662R1

Dear Dr. Umair,

We’re pleased to inform you that your manuscript has been judged scientifically suitable for publication and will be formally accepted for publication once it meets all outstanding technical requirements.

Within one week, you’ll receive an e-mail detailing the required amendments. When these have been addressed, you’ll receive a formal acceptance letter and your manuscript will be scheduled for publication.

An invoice will be generated when your article is formally accepted. Please note, if your institution has a publishing partnership with PLOS and your article meets the relevant criteria, all or part of your publication costs will be covered. Please make sure your user information is up-to-date by logging into Editorial Manager at Editorial Manager® and clicking the ‘Update My Information' link at the top of the page. For questions related to billing, please contact billing support.

If your institution or institutions have a press office, please notify them about your upcoming paper to help maximize its impact. If they’ll be preparing press materials, please inform our press team as soon as possible -- no later than 48 hours after receiving the formal acceptance. Your manuscript will remain under strict press embargo until 2 pm Eastern Time on the date of publication. For more information, please contact onepress@plos.org.

Kind regards,

Baochuan Lin, Ph.D.

Academic Editor

PLOS One

Additional Editor Comments (optional):

Reviewers' comments:

Reviewer's Responses to Questions

**Comments to the Author**

1. If the authors have adequately addressed your comments raised in a previous round of review and you feel that this manuscript is now acceptable for publication, you may indicate that here to bypass the “Comments to the Author” section, enter your conflict of interest statement in the “Confidential to Editor” section, and submit your "Accept" recommendation.

Reviewer #1: All comments have been addressed

Reviewer #2: All comments have been addressed

**********

2. Is the manuscript technically sound, and do the data support the conclusions?

The manuscript must describe a technically sound piece of scientific research with data that supports the conclusions. Experiments must have been conducted rigorously, with appropriate controls, replication, and sample sizes. The conclusions must be drawn appropriately based on the data presented.

Reviewer #1: Yes

Reviewer #2: Yes

**********

3. Has the statistical analysis been performed appropriately and rigorously?

Reviewer #1: Yes

Reviewer #2: Yes

**********

4. Have the authors made all data underlying the findings in their manuscript fully available?

The PLOS Data policy requires authors to make all data underlying the findings described in their manuscript fully available without restriction, with rare exception (please refer to the Data Availability Statement in the manuscript PDF file). The data should be provided as part of the manuscript or its supporting information, or deposited to a public repository. For example, in addition to summary statistics, the data points behind means, medians and variance measures should be available. If there are restrictions on publicly sharing data—e.g. participant privacy or use of data from a third party—those must be specified.

Reviewer #1: Yes

Reviewer #2: Yes

**********

5. Is the manuscript presented in an intelligible fashion and written in standard English?

PLOS ONE does not copyedit accepted manuscripts, so the language in submitted articles must be clear, correct, and unambiguous. Any typographical or grammatical errors should be corrected at revision, so please note any specific errors here.

Reviewer #1: Yes

Reviewer #2: Yes

**********

6. Review Comments to the Author

Please use the space provided to explain your answers to the questions above. You may also include additional comments for the author, including concerns about dual publication, research ethics, or publication ethics. (Please upload your review as an attachment if it exceeds 20,000 characters)

Reviewer #1: (No Response)

Reviewer #2: No major comments. All are addressed satisfactorily. However, please work on the quality of the figures.

**********

7. PLOS authors have the option to publish the peer review history of their article (what does this mean?). If published, this will include your full peer review and any attached files.

If you choose “no”, your identity will remain anonymous but your review may still be made public.

**Do you want your identity to be public for this peer review?**  For information about this choice, including consent withdrawal, please see our Privacy Policy.

Reviewer #1: No

Reviewer #2: **Yes:** Tesfaye Gelanew

**********

# Editor report: Acceptance letter

**DOI:** 10.1371/journal.pone.0353487.r004

**Authors:** Baochuan Lin

PONE-D-26-13662R1

PLOS One

Dear Dr. Umair,

I'm pleased to inform you that your manuscript has been deemed suitable for publication in PLOS One. Congratulations! Your manuscript is now being handed over to our production team.

At this stage, our production department will prepare your paper for publication. This includes ensuring the following:

* All references, tables, and figures are properly cited

* All relevant supporting information is included in the manuscript submission,

* There are no issues that prevent the paper from being properly typeset

You will receive further instructions from the production team, including instructions on how to review your proof when it is ready. Please keep in mind that we are working through a large volume of accepted articles, so please give us a few days to review your paper and let you know the next and final steps.

Lastly, if your institution or institutions have a press office, please let them know about your upcoming paper now to help maximize its impact. If they'll be preparing press materials, please inform our press team within the next 48 hours. Your manuscript will remain under strict press embargo until 2 pm Eastern Time on the date of publication. For more information, please contact onepress@plos.org.

You will receive an invoice from PLOS for your publication fee after your manuscript has reached the completed accept phase. If you receive an email requesting payment before acceptance or for any other service, this may be a phishing scheme. Learn how to identify phishing emails and protect your accounts at https://explore.plos.org/phishing.

If we can help with anything else, please email us at customercare@plos.org.

Thank you for submitting your work to PLOS ONE and supporting open access.

Kind regards,

PLOS ONE Editorial Office Staff

on behalf of

Dr. Baochuan Lin

Academic Editor

PLOS One
